# Ventilator-Associated Events Cost in ICU Patients Receiving Mechanical Ventilation: A Multi-State Model

**DOI:** 10.2478/jccm-2024-0016

**Published:** 2024-04-30

**Authors:** Alkmena Kafazi, Eleni Apostolopoulou, Vasiliki Benetou, Georgia Kourlaba, Christos Stylianou, Ioanna D Pavlopoulou

**Affiliations:** Faculty of Nursing, National and Kapodistrian University of Athens, Athens, Greece; Medical School, National and Kapodistrian University of Athens, Athens, Greece; Department of Nursing, University of Peloponnese, Tripoli, Greece; The 417 Army Equity Fund Hospital, Athens, Greece

**Keywords:** ventilator-associated events, cost, intensive care units, multi-state model, mechanical ventilation

## Abstract

**Introduction:**

Cost analysis is complicated by the fact that patients acquire infections during their hospital stay, having already spent time at risk without having an infection. Multi-state models (MSM) accounts for this time at risk treating infections as time-dependent exposures from ICU admission.

**Aim of the study:**

To estimate ventilator-associated events (VAEs) direct additional cost in ICU patients.

**Material and Methods:**

This was a prospective, observational study carried out for a two-year period in four medical-surgical ICUs of Athens, Greece. The sample consisted of adult patients who received mechanical ventilation for ≥4 days and were followed until discharge from the ICU or until death. CDC standard definitions were used to diagnose VAEs. To estimate VAEs additional length of stay (LOS), we used a four-state model that accounted for the time of VAEs. The direct hospital cost was calculated, consisting of the fixed and variable cost. The direct additional cost per VAEs episode was calculated by multiplying VAEs extra LOS by cost per day of ICU hospitalization.

**Results:**

In the final analysis were included 378 patients with 9,369 patient-days. The majority of patients were male (58.7%) with a median age of 60 years. Of 378 patients 143 (37.8%) developed 143 episodes of VAEs. VAEs crude additional LOS was 17 days, while VAE mean additional LOS after applying MSM was 6.55±1.78 days. The direct cost per day of ICU hospitalization was € 492.80. The direct additional cost per VAEs episode was € 3,227.84, € 885.56 the fixed and € 2,342.28 the variable cost. Antibiotic cost was € 1,570.95 per VAEs episode. The total direct additional cost for the two-year period was € 461,581.12.

**Conclusions:**

These results confirm the importance of estimating VAEs real cost using micro-costing for analytical cost allocation, and MSM to avoid additional LOS and cost overestimation.

## Introduction

Ventilator-associated events (VAEs) are consistently associated with increased duration of mechanical ventilation, hospital stay, ICU stay, and mortality. According to the Centers for Disease Control and Prevention (CDC) the mean incidence of VAEs ranged from 2 to 11.8 episodes per 1,000 ventilator-days and mortality in patients with VAEs was 31% [1]. According to previous studies from USA, VAEs prolongation in the duration of mechanical ventilation ranges from 7 to 14 days, in hospital stay from 4 to 16 days, in ICU hospitalization from 6 to 15 days, while attributable mortality ranges from 10% to 65.7% [2,3,4,5,6,7]. In Europe, the reported mean incidence of VAEs ranged from 10 to 107 episodes per 1,000 ventilator days, length of mechanical ventilation ranged from 4.1 to 13 days, hospital stay from 8.1 to 16 days, ICU stay from 8.2 to 14 days, while attributable mortality ranged from 22.2% to 37% [8,9]. However, despite the fact that VAEs are a frequent entity in mechanically ventilated patients, their economic implications remain unclear.

Biased results appear to be common in health care-associated infections (HCAIs) studies. A serious source of bias in ignoring the time dependence of HCAIs in analyses of length of stay (LOS) may lead to severely overestimation of HCAIs additional LOS [10,11]. Multi-state models (MSM) describe the occurrence of events over time as transitions between multiple states. They are an attractive tool to address the time dependence of HCAIs and offer further profound time-dependent insights into the burden of HCAIs [10,11].

When studying the burden of HCAIs not only the timing of HCAI acquisition but also the competing risks should be accounted for. A competing risk is an event whose frequency precludes the occurrence of the event under study. In our case, death and discharge alive without HCAIs are the competing risks for the occurrence of HCAIs, and discharge alive is the competing risk for in-hospital mortality. Ignoring competing risks may lead to an overestimation of HCAIs cumulative risk (competing risk bias) [10,11]. MSM allow the analysis of risks for all competing events and thus provide a suitable basis for the understanding and adequate statistical analysis of infections, as they take into account the competing risks of HCAIs and hospital mortality [10,11].

We aimed to estimate the direct additional cost – fixed and variable – of VAEs in adult ICU patients receiving mechanical ventilation using a MSM.

## Materials and methods

### Research design

A prospective observational study was carried out in four medical-surgical ICUs in Athens, Greece for the period January 2018 to December 2019. The sample consisted of all adult patients who received conventional mechanical ventilation for ≥4 days during surveillance. Follow-up of patients lasted from admission until discharge from the ICU or until death. In patients with more than one episode of VAE, only the first episode was considered. Exclusion criteria were age below 18 years, duration of mechanical ventilation less than 4 days, and confirmed infection on the day of ICU admission.

The diagnosis of VAEs was based on the criteria of the CDC standard definitions [12]. There are three levels of definition in the VAE algorithm: ventilator-associated conditions (VACs), infection-related ventilator associated complications (IVACs), and possible ventilator-associated pneumonia (PVAP). VACs refer to respiratory status deterioration defined as an increase in daily minimum PEEP ≥3 cm H_2_O or FiO_2_ ≥0.20 maintained for at least 2 days after a basic period (2 days) of stability or improvement. IVACs, along with the above criteria, include evidence of infection or inflammation defined as a white blood cell count ≥12,000 cells/mm^3^ or ≤4,000 cells/mm^3^, temperature >38 °C or <36 °C and a new antimicrobial prescription has been started and continued ≥4 days by the attending physician. PVAPs, in addition to the above criteria, include evidence of lower respiratory tract infection, defined as purulent respiratory secretions or positive culture or more strict criteria, including positive lung histopathological examination, positive pleural fluid culture and other tests, such as Legionella spp [12].

### Data collection

Data was collected daily from the patients’ files, from the forms of the nursing staff as well as from the micro-biological laboratory. For each mechanically ventilated patient, the daily minimum value of PEEP and FiO_2_ maintained for at least 1 hour during each calendar day was recorded. An interruption of mechanical ventilation for at least one day, followed by re-intubation or resumption of mechanical ventilation during the same hospitalization was recorded as a new episode of mechanical ventilation. For patients with VAEs, information was collected regarding mechanical ventilation conditions, amount and color of respiratory secretions, bronchial secretion cultures, antibiotic treatment, and chest radiographs.

### VAEs additional length of stay estimation

**Fig. 1.**
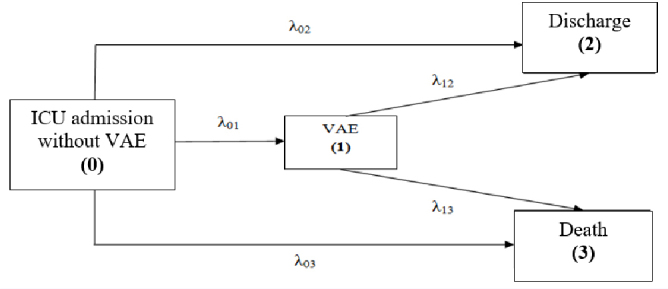
**Multi-state model with 4 states: 0, ICU admission without a VAE; 1, VAE (exposed); 2, discharge; and 3, death.** All patients entered into the initial state 0. Then the patient may acquire a VAE, moving to intermediate state 1, be discharged without a VAE, moving to state 2, or die without a VAE. Once acquired a VAE, the patient can move from state 1 to 2 or from 1 to 3. Hazard rates are denoted by k. with indices i and j indicating the states which they connect. VAE, Ventilator-Associated Event.

To estimate the additional LOS associated with VAEs, we used a MSM regarding VAE as a possible intermediate state between ICU admission and discharge or death. In the 4-state model used, admission (patients without VAEs) is state 0, VAE is state 1, and discharge (state 2) or death (state 3) indicates the end of ICU LOS. Patients in state 2 cannot enter state 3 (Figure 1).

We assumed that the distribution of time to event followed a recommended exponential distribution. For cases in a given state up to a specified time, the hazard (λ) approximates the probability of moving from one state to another in a short interval, divided by the length of the interval. Based on the 5 hazards from the MSM, it is possible to quantify risk and clinical outcomes of VAE in terms of mortality and LOS (Table 1) [11].

**Table 1.** Multi-state analysis of Ventilator-Associated Events

**Constant hazards from MSM**	
**VAE hazard**	λ_01_
Discharge hazard without VAE	λ_02_
Death hazard without VAE	λ_03_
Discharge hazard with VAE	λ_12_
Death hazard with VAE	λ_13_

**Occurrence of VAE**	
Incidence rate of VAE per patient day	λ_01_
Mortality due to VAE	
ICU mortality	(λ_01_λ_13_ / λ_01_λ_02_λ_03_) + λ_03_ / λ_01_λ_02_λ_03_
Hazard ratio of VAE (death)	λ_13_ / λ_03_
Hazard ratio of VAE (discharge)	λ_12_ / λ_02_
Attributable mortality of VAE	(λ_02_λ_13_ − λ_03_λ_12_) / (λ_12_ + λ_13_) (λ_02_ + λ_03_)
Population attributable fraction for mortality	[λ_01_ (λ_02_λ_13_ − λ_03_λ_12_)] / (λ_02_ + λ_03_) (λ_03_λ_1_ + λ_01_λ_13_), λ_1_ = λ_12_ + λ_13_

MSM, multi-state model; VAE, ventilator-associated event; ICU, intensive care unit.

Since the change in LOS due to VAEs is caused by the days after VAEs are acquired, it is necessary to consider the timing of VAEs. Based on the patients’ pathway across available model-states transitions, the MSM provides a weighted average of the LOS. The mean difference in LOS was calculated for each day as the difference between the estimated LOS given whether the intermediate state (VAEs) had been achieved or not by that day. The overall change in LOS was calculated as a weighted average of these quantities, with the weighting determined by the observed distribution of time to onset of VAEs [13].

All patients were followed up until discharge or death in ICU; there was no administrative censoring. VAEs could be one of the following events: VACs, IVACs or PVAPs. Each of the patients was infected from only one of these. The additional LOS is presented overall (VAEs) and stratified by VAEs event (VACs, IVACs or PVAPs). Also, the additional LOS was calculated separately for patients with VAEs who were discharged alive, as well as for patients with VAEs who died in the ICU.

### Economic evaluation

The direct cost of ICU hospitalization was calculated using the bottom-up method, which constitutes a microeconomic approach of analytical distribution of costs for each hospitalized patient according to the use of resources [14]. Direct hospital costs, consisting of fixed and variable cost, were calculated for each patient.

The daily fixed cost was derived from the payroll of the staff and the ICUs operating and maintenance costs. These costs were obtained from data provided by the Greek Ministry of Health based on the annual cost reports of the hospitals participating in the study [15]. ICU LOS was recorded prospectively for each patient and the number of days in the ICU was used to calculate the fixed cost of hospitalization in the ICU. Fixed cost was calculated by multiplying ICU LOS by the corresponding daily ICU costs.

Variable costs were derived from daily costs of antibiotics and other medications, enteral and parenteral nutrition, blood products, medical supplies, and laboratory/imaging tests. The cost of medication was calculated by multiplying the daily dose of each drug by the days of medication for each patient and then multiplying by the price of the drug per recorded unit (gr, ml, etc.). Hospital costs of drugs, enteral and parenteral nutrition, as well as transfusion costs were derived from relevant data from the Ministry of Health [16]. In order to record the exact dosage of antibiotics and other drugs, a daily review of patient files was performed. The cost of the medical supplies was obtained from the official “Observe Net” website of the Health Supplies Committee [17], which is the medical supplies price observatory for Greek public hospitals. Medical supplies included were endotracheal tubes, tracheostomy tubes, central venous catheters, bladder catheters, pressure ulcer pads, and enteral and parenteral feeding devices. The costs of diagnostic tests were obtained from the official website of the National Organization for Healthcare Services Provision [18]. These costs were then multiplied by the number of diagnostic tests performed on each patient. Diagnostic tests included are electrocardiogram, electroencephalogram, radiographs, ultrasound, CT and MRI scans, as well as microbiological tests (cultures).

The direct additional cost per VAEs episode was calculated by multiplying VAEs additional LOS by the cost per day of ICU hospitalization. The cost per ICU hospitalization day was calculated by dividing the sum of the direct ICU hospitalization costs for all patients by the sum of the ICU days for all these patients. The total direct additional cost of VAEs over the 2-year period was calculated by multiplying the number of VAE cases by the derived additional LOS and the estimated costs per ICU bed day.

### Statistical analysis

Categorical variables were expressed as absolute (N) and relative frequencies (%) and differences between the two groups were compared using the χ^2^ test or the Fisher exact test, where appropriate. Continuous variables were expressed as median and interquartile range, and differences between groups were compared using the nonparametric Mann-Whitney U-test. The Kolmogorov-Smirnov test was used to test for normality. Descriptive analysis of the data was performed using statistical packages for social science version 22 (SPSS Inc., Chicago, IL).

The mean additional LOS of VAEs was calculated using MSM, taking into account the time dependence of VAEs and thus avoiding time-dependent bias. Transitions between states were determined by time-varying hazards, which were estimated using the Aalen-Johansen estimator. The expected LOS associated with VAEs was computed by a function of these transition probabilities. We calculated the standard error for extra LOS by bootstrap sampling using 1,000 replicates. The bias-correlated and accelerated confidence intervals (BCa CIs) were estimated with the method of DiCiccio and Efron [19]. All analyses were performed with R (version 4.2.0) via RStudio (2022.02.2 – 485) using the *etm* and *mvna* packages.

### Research ethics

The present study responded to the fundamental principles of ethics. Complete confidentiality was maintained regarding the data collected, anonymity secured, and the research protocol was approved by the Hospitals’ Scientific Committees, which has waived the need for informed consent.

## Results

### Baseline characteristics

During the 2-year study period, 500 patients were hospitalized for ≥4 days under mechanical ventilation in 4 medical-surgical ICUs; 122 were excluded because of confirmed infection on the day of admission. In the final analysis, 378 patients with 9,369 days of ICU hospitalization were included. The characteristics of patients with and without VAEs are shown in Table 2.

**Table 2.** The characteristics of patients with and without ventilator-associated events

	**VAE**		**P Value**

	**Yes** **N = 143, %**	**No** **N= 235, %**	
Gender			0.451
Male	88 (61.5)	134 (57)	
Age, years (Median, Interquartile range)	59 (46–73)	61 (45–74)	0.649

**Type of patient**			
Medical	74 (51.7)	115 (48.9)	0.671
Surgical	69 (48.3)	120 (51.1)	

**McCabe score**			
Non-fatal disease	49 (34.3)	114 (48.5)	0.007
Ultimately fatal disease	16 (11.2)	20 (8.5)	0.470
Rapidly fatal disease	78 (54.5)	101 (43)	0.034

**Admission diagnosis**			
Trauma	42 (29.4)	43 (18.3)	0.016
Neurological disease	30 (21)	42 (17.9)	0.500
Pulmonary disease	33 (23.1)	35 (14.9)	0.053
Post-operative observation	18 (12.6)	66 (28.1)	0.000
Cardiovascular disease	11 (7.7)	26 (11.1)	0.372
Malignancy	5 (3.5)	17 (7.2)	0.175
Other (burn, poisoning)	4 (2.8)	6 (2.6)	1.000
Death	63 (44.1)	55 (23.4)	0.000

VAE, ventilator-associated event.

### The rates of VAEs

In total, 143 of 378 patients (37.8%) developed 143 VAEs (83 VACs, 44 IVACs and 16 PVAPs). Overall, 72 VAEs (50.3%) had an onset date on or after day 5 of mechanical ventilation. The rate of VAEs was 32.36 episodes per 1,000 ventilator-days. The highest incidence rate (14.30 episodes per 1,000 ventilator-days) was observed in VAC, followed by IVAC (6.85 episodes per 1,000 ventilator-days) and PVAP (2.30 episodes per 1,000 ventilator-days).

### Multi-state model results

The risk of VAE (λ_01_) was 2.6 times greater than the competing risk of death (λ_03_) and 0.8 times less of competing risk of discharge without VAE (λ_02_). For all types of VAE it appears that λ_02_ was greater than λ_12_. This means that patients without VAE had a higher daily discharge probability, compared to patients with VAE. Also, λ_13_ was greater than λ_03_. This means that patients with VAE had a higher daily risk of death in the ICU, compared to patients without VAE (Table 3).

**Table 3.** Multi-state model constant hazards

**Constant hazards (95% CI)**	**VAE**	**VAC**	**IVAC**	**PVAP**
λ_01_ VAE hazard	0.026 (0.022–0.030)	0.011 (0.009–0.014)	0.005 (0.003–0.007)	0.001 (0.001–0.002)
λ_02_ Discharge hazard without VAE	0.033 (0.028–0.038)	0.029 (0.026–0.034)	0.028 (0.025–0.032)	0.028 (0.025–0.032)
λ_03_ Death hazard without VAE	0.010 (0.007–0.013)	0.011 (0.009–0.014)	0.012 (0.010–0.014)	0.012 (0.010–0.014)
λ_12_ Discharge hazard with VAE	0.020 (0.016–0.025)	0.021 (0.015–0.027)	0.021 (0.014–0.031)	0.014 (0.006–0.027)
λ_13_ Death hazard with VAE	0.016 (0.012–0.020)	0.016 (0.011–0.022)	0.014 (0.091–0.023)	0.018 (0.008–0.033)

CI, confidence interval; VAE, ventilator-associated event; VAC, ventilator-associated condition; IVAC, infection-related ventilator-associated complication; PVAP, possible ventilator-associated pneumonia.

The overall ICU mortality was 31.2% and the competitive risk of ICU discharge alive was 68.8% (260/378). Death hazard ratio was 1.582 (95% CI: 1.101–2.278), which means that patients with VAE had a 58.2% increased risk of death, compared to patients without VAE. The discharge hazard ratio was 0.614 (95% CI: 0.509 – 0.960), which means that patients with VAE had a 38.6% reduced probability of discharge, compared to patients without VAE (Table 4).

**Table 4.** Death and discharge hazard ratio for patients with VAE

**Types of VAE**	**Death hazard ratio (95% CI)**	**Discharge hazard ratio (95% CI)**
VAC	1.401 (0.938–2.063)	0.704 (0.509–0.960)
IVAC	1.223 (0.721–1.984)	0.755 (0.494–1.116)
PVAP	1.471 (0.701–2.788)	0.493 (0.233–1.044)
VAEs	1.582 (1.101–2.278)	0.614 (0.470–0.797)

CI, confidence interval; VAE, ventilator-associated event; VAC, ventilator-associated condition; IVAC, infection-related ventilator-associated complication; PVAP, possible ventilator-associated pneumonia.

The attributable mortality of VAEs was 21.2%. PVAPs had the highest attributable mortality (26.3%), followed by VACs (15.7%), and IVACs (10.0%). The population attributable fraction was 25.6%. By type of VAE it appears that overall mortality in the study population would have been reduced by 11% in the absence of VAC and by 3.6% and 2.1% in the absence of IVAC and PVAP, respectively.

The crude additional LOS for VAEs was 17 days (27.5 days in patients with VAEs versus 10.5 days in patients without VAEs, p<0.001).

**Fig. 2.**
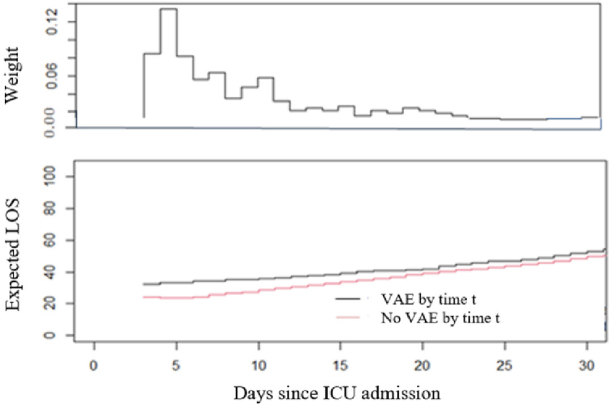
**Plot of the expected LOS in ICU.** The black line shows that a VAE has occurred, and the red line indicates a lack of VAE up to 30 days after ICU hospitalization. The weights (distribution of the time to VAE) are illustrated in the upper plot. VAE, ventilator-associated event; LOS, length of stay.

Figure 2 illustrates the estimated LOS of patients with VAE still hospitalized, versus those still hospitalized and without VAE, for each day of the study interval up to 30 days after ICU admission. For each day under study, the expected LOS of patients with VAE appears longer than the expected LOS of patients still without VAE on the same day. These differences resulted in an excess LOS of 6.55 days (95% BCa CI: 2.76–9.73); 3.30 additional ICU days regarding death; and 3.25 additional days regarding discharge (Table 5).

**Table 5.** Mean additional length of stay associated with VAEs

	**Additional ICU days regarding discharge ± SE**	**95% BCa CI**	**Additional ICU days regarding death ± SE**	**95% BCa CI**	**Total additional ICU days ± SE**	**95% BCa CI**
VAC	4.50 ± 1.93	1.14–8.87	2.43 ± 1.22	0.21–4.81	6.93 ± 2.38	2.45–11.83
IVAC	1.92 ± 1.81	−0.93–6.18	5.35 ± 2.45	0.85–10.48	7.27 ± 2.94	1.68–13.08
PVAP	3.99 ± 2.65	−0.25–10.30	7.09 ± 5.38	−1.21–18.57	11.08 ± 5.65	0.60–22.05
All VAEs	3.25 ± 1.37	0.71–6.08	3.30 ± 1.17	1.10–5.64	6.55 ± 1.78	2.76–9.73

BCa CI, Bias-corrected and accelerated confidence interval; SE, standard error; VAE, ventilator-associated event; VAC, ventilator-associated condition; IVAC, infection-related ventilator-associated complication; PVAP, possible ventilator-associated pneumonia.

The direct cost per day of ICU hospitalization was €492.80. Fixed and variable costs per ICU day were €135.20 and €357.60, respectively. Regarding the variable cost, the cost of antibiotics was higher (€239.84) followed by the cost of other drugs (€102.06), diagnostic tests (€7.80), medical equipment (7.26 €) and transfusion (0.64 €).

The direct additional cost per VAE episode was €3,227.84 (Table 6). Variable cost accounts for 73% (2,342.28 / 3,227.84), while fixed costs account for 27% (885.56 / 3,227.84) of direct costs. With reference to the variable cost subcategories, antibiotics account for the largest percentage (67.1%) followed by other drugs (28.5%). The total direct additional cost for the 2-year period was €461,581.12 for VAEs; €283,453.30 for VACs; €157,636.60 for IVACs; and €87,363.52 for PVAPs.

**Table 6.** The direct additional cost per VAE episode

	**Direct additional cost per VAE episode, €**		
	**Additional cost regarding death**	**Additional cost regarding discharge**	**Total additional cost**
VAC	1,197.50	2,217.60	3,415.10
IVAC	2,636.48	946,17	3,582.65
PVAP	3,493.95	1,966.27	5,460.22
All VAEs	1,626.24	1,601.60	3,227.84

VAE, ventilator-associated event; VAC, ventilator-associated condition; IVAC, infection-related ventilator-associated complication; PVAP, possible ventilator-associated pneumonia.

## Discussion

The use of appropriate statistical methods to estimate the additional LOS of VAEs that increases the associated costs has not been reported, and this study is the first to estimate the direct (fixed and variable) costs of VAEs in the ICU using a MSM to avoid time-dependent bias and overestimation of LOS.

Our findings show that VAEs are a significant problem in the 4 ICUs studied. The higher incidence of VAEs (32.36 episodes per 1,000 ventilator-days) compared to CDC data (4.48 episodes per 1,000 ventilator-days) [1], could be attributed to the lack of a well-organized surveillance system and infection control program, insufficient numbers of trained infection control staff and reduced resources for infection control. Also, the observed difference in the incidence of VAEs compared to previous studies (6–107 episodes per 1,000 ventilator-days) may be attributed to the different denominator used to calculate the incidence, as shown by studies that included all mechanically ventilated patients [2, 3, 5], or only patients with at least 48 hours [7], 4 days [20], or 5 days on the ventilator [9].

Interestingly, in hospital epidemiology the MSM is a useful tool to study the link between HCAIs and mortality. This model not only accounts for the time dependence of the acquisition of VAEs, but also differentiates between the competing risks of discharge alive and death. Ignoring the risk of discharge could lead to biased estimates [21]. Our findings show that patients with VAEs are associated with an increased risk of death by 58.2% and a reduced risk of discharge by 38.6%.

Also, the application of the MSM avoids the overestimation of the additional LOS, and therefore the additional cost, as it takes into account the bias of the time-dependent exposure of VAEs. In the present study, the additional LOS for VAEs was 6.5 days, leading to 930 additional ICU days used for patients with VAE (143 × 6.5 = 930). As according to the World Health Organization 35–70% of HCAIs are preventable [22], a commensurate reduction in the incidence of VAEs would lead to a benefit of 326 to 651 free ICU bed-days after implementing an effective VAE prevention program. The additional LOS for VAEs varies between studies, which calculated only the crude additional LOS (6–21.9 days) [3, 6, 8, 9]. These findings highlight the use of VAE as a time-dependent exposure and confirm the importance of accurate results using appropriate statistical methods [23, 24].

Based on the exact estimate of the additional LOS we calculated the direct additional cost of VAEs. Our findings are difficult to compare, as the relevant literature is quite limited. The largest direct additional cost per VAE episode in the study of He et al. ($6,775.49) [20] and Harris et al. ($14,557) [25], compared to our study ($3,227.84) may be attributed to the different costing methodology of the previous authors, which were based on hospital charges than the real cost [20] and the use of a conservative costing model of the previous researchers, who calculated the costs from the day after the appearance of VAEs and were limited to seven days later [25].

By type of VAE, the highest cost of PVAPs (€5,460.22) can be attributed to the longest additional LOS in these patients (11.08 days). Our findings are similar with He et al. [20] who report that costs were greater for PVAPs ($15,012.59), but contradict the study by Harris et al. reporting that the largest cost was for IVACs ($20,278) [25]. These differences can be attributed to several factors: costs for same services vary between countries, ICUs are not standard in size and have different staff/patient ratios, treatment options differ, thus affecting patient costs, and most importantly, there is no standardized methodology for costing HCAIs in the ICU and methods vary between studies.

It is worth emphasizing that the hospitals were charged €461,581.12 just for the diagnosis and treatment of the 143 VAE episodes. As 35–70% of VAEs are preventable [22], the economic benefit to the hospitals in our study would be from €161,553.39 to €323,106.78, showing the effectiveness of a VAE prevention package.

### Limitations

Our results should be interpreted in the context of some possible limitations. First, this study was performed in 4 medical-surgical ICUs, and our results may not be generalizable to other hospitals, given differences in staffing, patient population, and available resources and structures. Second, the economic consequences of VAE related morbidity and mortality from a societal perspective (e.g. lost productivity) were not considered. Because of this perspective, the time horizon of the analysis is limited to the period of hospitalization in the ICU. However, VAEs impose a significant burden on other environments as well. After leaving the ICU, patients are transferred to hospital clinics. In addition to the costs to the health care sector, there may be costs to the patient and their family due to lost work hours. Further analysis could therefore be considered to extend this perspective beyond the ICU [26].

Despite the limitations, our study has several strengths as it is a prospective study, which presents an accurate mapping of the clinical and economic impact of VAEs, based on CDC standard definitions and protocols for VAEs diagnosis and patient monitoring [12]. Our findings highlight the problem of VAEs and although they reflect the Greek ICU situation they are useful for other countries as well. Some follow up after the results are sent to the managers or authorities will be profitable in order to see what we can do to reduce these costs.

In a time of cost containment, estimating the financial burden of VAEs is a key tool for infection control professionals competing for resources [27]. It has become clear that a more structured approach to costing VAEs in the ICU is needed to enable a better comparison between published data [28]. However, cost estimation is not straightforward. The methodology we used differs from those used in previous studies. First, we used a MSM, which provides a conceptual framework for appropriate statistical analyses, such as time-dependent exposure, competing risks, and other issues, such as estimating attributable mortality and LOS [11]. Second, our method approaches micro-costing techniques, which allow the calculation of the total cost of each species and each patient, knowing the exact resource consumption for each patient [27]. We relied on a methodologically rigorous cost analysis, including not only fixed costs, but also the costs of antibiotics, other drugs, transfusions, diagnostic tests, and medical supplies. Additionally, we used actual costs incurred by the hospital rather than charges, as this is a more accurate measure [29].

## Conclusion

Our results confirm the importance of MSM for the estimation of the additional LOS, the removal of patients with infection at admission, and micro-costing for the analytical distribution of costs. The forecast of the benefit of the studied ICUs from 326 to 651 days of free ICU beds and from €161,553.39 to €323,106.78, mainly in a period of great pressure for free ICU beds experienced by our country, confirms the need to implement VAE prevention measures.

As a final conclusion, our study highlights the commitment of clinical and infection control staff to the goal of “zero tolerance for HCAIs” and of hospital administrations and government officials to increase resources in infection control to reduce the burden of VAEs, for safety and quality care of ICU patients in our country at the lowest possible cost.
